# Substandard anti-malarial drugs in Burkina Faso

**DOI:** 10.1186/1475-2875-7-95

**Published:** 2008-05-27

**Authors:** Maike Tipke, Salou Diallo, Boubacar Coulibaly, Dominic Störzinger, Torsten Hoppe-Tichy, Ali Sie, Olaf Müller

**Affiliations:** 1 Department of Tropical Hygiene and Public Health, Ruprecht-Karls-University Heidelberg, Im Neuenheimer Feld 324, 69120 Heidelberg, Germany; 2 Centre de Recherche en Santé de Nouna (CRSN), Nouna, POB 2, Burkina Faso; 3 University Pharmacy, Ruprecht-Karls-University Heidelberg, Im Neuenheimer Feld 670, 69120 Heidelberg, Germany

## Abstract

**Background:**

There is concern about an increasing infiltration of markets by substandard and fake medications against life-threatening diseases in developing countries. This is particularly worrying with regard to the increasing resistance development of *Plasmodium falciparum *against affordable anti-malarial medications, which has led to a change to more expensive drugs in most endemic countries.

**Methods:**

A representative sample of modern anti-malarial medications from licensed (public and private pharmacies, community health workers) and illicit (market and street vendors, shops) sources has been collected in the Nouna Health District in north-western Burkina Faso in 2006. All drugs were tested for their quality with the standard procedures of the German Pharma Health Fund-Minilab. Detected low standard drugs were re-tested with European Pharmacopoeia 2.9.1 standards for disintegration and ultraviolet-visible spectroscopy at the laboratory of the Heidelberg University for confirmation.

**Results:**

Overall, 86 anti-malarial drug samples were collected, of which 77 samples have been included in the final analysis. The sample consisted of 39/77 (50%) chloroquine, 10/77 (13%) pyrimethamine-sulphadoxine, 9/77 (12%) quinine, 6/77 (8%) amodiaquine, 9/77 (12%) artesunate, and 4/77 (5%) artemether-lumefantrine. 32/77 (42%) drug samples were found to be of poor quality, of which 28 samples failed the visual inspection, nine samples had substandard concentrations of the active ingredient, four samples showed poor disintegration, and one sample contained non of the stated active ingredient. The licensed and the illicit market contributed 5/47 (10.6%) and 27/30 (90.0%) samples of substandard drugs respectively.

**Conclusion:**

These findings provide further evidence for the wide-spread existence of substandard anti-malarial medications in Africa and call for strengthening of the regulatory and quality control capacity of affected countries, particularly in view of the now wider available and substantially more costly artemisinin-based combination therapies.

## Background

Malaria remains the globally most important parasitic disease. Out of the approximately one million deaths caused by malaria each year, the great majority occurs in children under the age of five years living in sub-Saharan Africa (SSA) [1].

Malaria, if treated early with effective drugs, is fully curable. However, most of the affected children live in remote and poor communities with low access to functioning modern health services [1,2]. As a result, children are either not treated at all, receive traditional treatment of uncertain efficacy, or are treated with drugs primarily bought at the informal drug sector [1,3].

In recent years, increasing numbers of substandard and fake medications were detected in the international markets, but precise figures of the global situation are lacking. It is estimated that more than 10% of the globally traded medicines are counterfeits [4,5]. In developing countries, where regulatory and control mechanisms are weak, people are at highest risk to purchase substandard medications [4,6,7]. Seiter concluded in 2005: "Pharmaceutical products are attractive candidates for illegal trade, especially in developing countries. They are easily transportable, have high value per unit, and most importantly, their quality cannot be assessed readily by lay persons or even experts without the aid of a quality testing laboratory" [8]. Drugs to treat infectious diseases, like malaria, pneumonia or diarrhoea, are frequently a target of criminal action [5,9,10].

After the marketing of the artemisinin-based combination therapy (ACT) against malaria in Asia, these costly drugs were found to be counterfeit in 38% and 53% in two studies conducted in different countries of south-east Asia [11,12]. In Cambodia, for example, it was shown that fake artesunate was sold by 71% of local drug vendors [13]. Globally it is estimated that more than US$ 30 billion per year are earned by the overall trade of substandard and counterfeit drugs and this will probably increase to US$ 75 billion by the year 2010 [8,14].

The intake of fake medicaments can lead to life-threatening consequences [5,7,11]. Especially for diseases carrying a high mortality if left untreated, like malaria, substandard drugs will raise death rates. Furthermore, such drugs can lead to numerous adverse drug effects due to under-dosing, over-dosing, and unexpected or toxic substances [5,11]. Moreover, it will influence the economic welfare of patients, health systems, and drug companies that produce genuine products [11]. Finally, it will most likely increase the risk of selection and spreading of drug resistance [11]. Fake drugs, furthermore, can lead to biased data on drug resistance or drug efficacy studies [9,15].

The World Health Organization (WHO), thus, recommended taking action against substandard drugs at the individual country level [16]. This recommendation is supported by the German Pharma Health Fund (GPHF) that designed an affordable laboratory kid for screening drugs even under tropical conditions. The GPHF-Minilab is working with widely available reagents and can also be run without electricity [17-21]. The GPHF-Minilab operates in a four step procedure, including two physical methods (visual inspection and disintegration test) and two chemical methods (qualitative colour reaction test and semi-quantitative thin-layer chromatography) [20].

The aim of this study was to evaluate the presence of substandard anti-malarial drugs in the public, private, and informal markets of Burkina Faso.

## Methods

### Study area

The study was carried out in the Nouna Health District (NHD), a rural, multi-ethnic area of north-western Burkina Faso, West Africa. Most of the estimated 280,000 inhabitants of NHD are living under self-subsistence conditions. Malaria is the main cause of morbidity and mortality in the NHD as in the whole of Burkina Faso [22]. Formal health services in NHD comprise of 25 primary health care facilities, which are covering the needs of a variable number of villages, and the district hospital in Nouna town [2]. NHD is holoendemic for malaria with elevated transmission rates during and shortly after the rainy season, which typically lasts from June to October [22].

### Study design and procedures

This cross-sectional study was carried out during the rainy season of 2006. Drugs were sampled in August and September which are known to be the months of most intense drug sale in this area of West Africa [23].

Anti-malarial drugs were collected from a sample of 16 villages (based on specific information from the results of a representative anti-malarial drug provider study conducted in early 2006 in the whole of NHD), and in Nouna town. After identification of all points of drug sale in these villages, anti-malarial drug samples were purchased from markets, street vendors, shops, private pharmacies, community health workers, and the governmental pharmacies attached to the peripheral health centres. Each market place was defined as one point of sale and the drug collection was carried out on respective market days. For the analysis, private pharmacies, community health workers, and the health centre and hospital pharmacies were defined as licensed market, while markets, street vendors, and shops were summarized as illicit market. The sample consisted of tablets and capsules of chloroquine, amodiaquine, sulphadoxine/pyrimethamine, quinine, artesunate and, artemether-lumefantrine.

Drugs were purchased by one field worker who behaved like a normal client. Each sample of a drug was labelled with an identification number and put into a plastic bag without any further manipulation. A drug collection sheet, which provided information on the date, place, and conditions of purchase, the name of the drug indicated by the vendor and the name stated on the product, the active ingredient and the price, was filled in immediately. After purchase, all drug samples have been stored in a dark, dry and air conditioned place inside the laboratory building of the *Centre de Recherche en Santé de Nouna *(CRSN).

At the time-point of the study no artemisinin drugs or ACT were available in the NHD, except in the two private pharmacies in Nouna town. To get a broader impression on the quality of artemisinin drugs or ACT in Burkina Faso, a convenience sample of such products was taken from market places and private pharmacies in randomly selected quarters of Ougadougou, the capital of Burkina Faso.

### Laboratory work

The initial testing of all drug samples was performed at the laboratory of the CRSN with the German Pharma Health Fund (GPHF)-Minilab. Two physical methods (visual inspection and disintegration test) and two chemical methods (qualitative colour reaction test and semi-quantitative thin-layer chromatography) were performed according to the existing GPHF-Minilab standards. All data was noted down in the specific "reporting forms" of the GPHF-Minilab.

GPHF-Minilab procedures were performed by the two investigators (MT and SD). In case a sample failed the visual inspection by one of the investigators it was re-examined by the second investigator for confirmation. Furthermore, in cases of doubt external aid was consulted (e.g. internet research on manufactures information and figures provided on homepages). Drug samples which failed the colour reaction by the first investigator, i.e. none of the stated and expected active ingredient was present, were retested in a second run by the respectively second investigator for confirmation. The aim of this procedure was to confirm the coherence of the stated and actually present active ingredient. Further investigations to determine potential other present active ingredients, which were not indicated, were therefore, not performed.

Drug samples that failed the disintegration test or semi-quantitative thin-layer chromatography in the GPHF-Minilab testings, were sent to Germany, where confirmatory tests took place at the laboratory of the pharmacy of the Heidelberg University. The confirmatory disintegration tests were performed according to European Pharmacopoeia 2.9.1 [24]. The quantification of the active ingredient of respective samples was done with the ultraviolet-visible spectroscopy [25,26]. The German investigators were blinded to the origin of respective samples.

### Data analysis

All data were transferred into a Microsoft Excel database. The descriptive analysis was done by SPSS 12.0 for Windows.

### Ethical aspects

The study is part of the A8 study of the SFB 544, which has been approved by the local Ethical Committee of the CRSN, Burkina Faso, and by the Ethical Committee of the Heidelberg University. The specific study procedures were discussed and approved by the responsible authorities of the NHD. At each point of sale, not more than 50% of the total amount of available malaria drugs was purchased.

## Results

**Figure 1:**
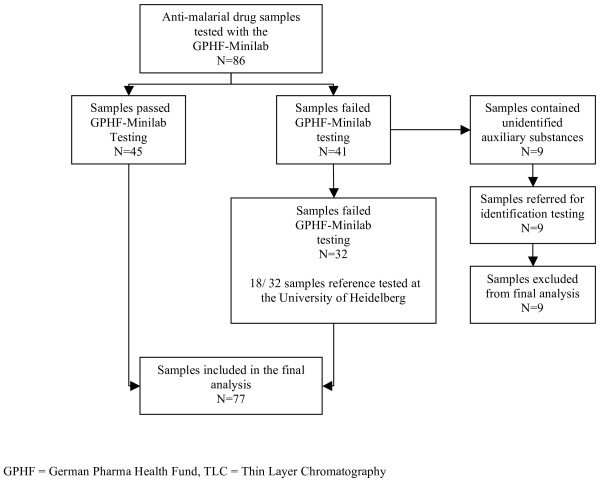
Flow chart of study sample.

A total of 86 anti-malarial drug samples have been collected, 79 came from the NHD and seven from Ouagadougou (Figure 1). Of these, 48/86 (56%) were chloroquine samples, 6/86 (7%) were amodiaquine samples, 10/86 (12%) were pyrimethamine-sulphadoxine samples, 9/86 (10%) were quinine samples, and 13/86 (15%) were artemisinin or ACT samples (of those, six were from NHD and seven from Ouagadougou).

Overall, 18/86 (21%) samples, which had failed the GPHF-Minilab disintegration and/or semi-quantitative thin-layer chromatography tests, were re-tested for confirmation. Another 9/86 (10%) drug samples showed tailing phenomena in the GPHF-Minilab semi-quantitative thin-layer chromatography. Tailing phenomena indicate possible auxiliary substances in the respective drug samples (all nine samples were chloroquine). These samples were also referred for further investigation to the pharmacy of the University of Heidelberg. Here it was concluded that these phenomena were most probably caused by povidone, which are widely used as excipient in tablets. As it cannot be excluded that other unknown substances have caused the tailing phenomena, these nine samples were excluded. Thus, 77 anti-malarial drug samples were finally included in the analysis (Figure 1, Table 1). Of these, 47/77 (61%) were from the licensed market and 30/77 (39%) were from the illicit market (Table 2).

**Table 1:** Origin of anti-malarial drugs included into the final analysis

	**NHD except Nouna town**	**Nouna town**	**Ouagadougou**	**total**	**%**
**Chloroquine**	31	8	0	39/77	50.6
**Artesunate**	0	4	5	9/77	11.7
**Lumefantrine/Artemether**	0	2	2	4/77	5.2
**Sulphadoxine/Pyrimethamine**	7	2	0	10/77	13.0
**Quinine**	6	2	0	9/77	11.7
**Amodiaquine**	3	3	0	6/77	7.8
**Total**	47	23	7	77/77	100

NHD = Nouna Health District

**Table 2:** Points of sale and numbers of anti-malarial drugs included in the sample

	**NHD except Nouna town**	**Nouna town**	**Ouagadougou**	**In total**	**%**
**Public health facility**	18	4	0	22/77	28.6
**Community health worker**	3	0	0	3/77	3.9
**Private pharmacy**	4	13	5	22/77	28.6
**Shop**	1	3	0	4/77	5.2
**Street vendor**	0	1	0	1/77	1.3
**Market place**	21	2	2	25/77	32.5
**Total**	47	23	7	77/77	100

NHD = Nouna Health District

In total, 32/77 (41.6%) anti-malarial drug samples were found to be of substandard quality, of which 28/32 (88%) samples failed the visual inspection. These samples have been chloroquine, 23/28 (82%); sulphadoxine/pyrimethamine, 4/28 (14%); and artesunate, 1/28 (4%). 4/32 (13%) samples were found to be of poor disintegration (three chloroquine, one sulphadoxine/pyrimethamine) (Table 3).

**Table 3:** Number (%) of failed samples listed by the active ingredient and test

	**Visual inspection**	**Disintegration test**	**Colour reaction**	**Thin-layer chromatography and ultraviolet-visible spectroscopy**	**Total**
**Chloroquine**	23/39 (59.0%)	3/39 (7.7%)	0/39	5/39 (12.8%)	24/39* (61.5%)
**Artesunate**	1/9 (11.1%)	0/9	0/9	0/9	1/9 (11.1%)
**Lumefantrine/Artemether**	0/4	0/4	0/4	0/4	0/4
**Sulphadoxine/Pyrimethamine**	4/10 (40.0%)	1/10 (10.0%)	1/10 (10.0%)	1/10 (10.0%)	4/10* (40%)
**Quinine**	0/9	0/9	0/9	3/9 (33.3%)	3/9 (33.3%)
**Amodiaquine**	0/6	0/6	0/6	0/6	0/6
**Total**	28/77 (36.4%)	4/77 (5.2%)	1/77 (1.3%)	9/76 (11.8%)	32/77 (41.6%)

* Some samples failed more than one test

One of 32 (3%) samples (sulphadoxine/pyrimethamine) failed the colour reaction, indicating neither sulphadoxine nor pyrimethamine was present in this sample. This sample, thus, was excluded from the following semi-quantitative thin-layer chromatography tests and ultraviolet-visible spectroscopy. Here, 9/31 (29%) anti-malarial drug samples with substandard concentrations of the respective active ingredient were detected. Of these, 5/9 (55.6%) were chloroquine, 3/9 (33.3%) were quinine, and 1/9 (11.1%) were sulphadoxine/pyrimethamine (Table 4).

**Table 4:** Real value analysis of samples with substandard concentrations of the active ingredient

**Active ingredient**	**Place of purchase**	**Real value analysis (mg)**	**Difference**
Chloroquine Phosphate (100 mg)	Private pharmacy	min: 89,94	10%
		max: 94,08	6%
		mid: 91,57	8%
Chloroquine Phosphate (100 mg)	marked place	min: 87,32	13%
		max: 91,07	9%
		mid: 88,68	11%
Chloroquine Phosphate (250 mg)	marked place	min: 180.16	28%
		max: 221.31	11%
		mid: 200.93	20%
Chloroquine Phosphate (250 mg)	marked place	min: 188,01	25%
		max: 198,67	20%
		mid: 193.34	23%
Chloroquine Phosphate (250 mg)	marked place	min: 188,38	25%
		max: 193,79	22%
		mid: 191.08	24%
Sulfadoxine/Pyrimethamine (500/25 mg)	marked place	only TLC	> 20%
		performed	> 20%
Quinine Sulfate (300 mg)	public health facility	min: 267,42	11%
		max: 291,95	3%
		mid: 277.37	7%
Quinine Sulfate (300 mg)	public health facility	min: 176,11	41%
		max: 286,60	4%
		mid: 237.04	21%
Quinine Sulfate (300 mg)	Privat pharmacy	min: 251,11	16%
		max: 276,09	8%
		mid: 266.14	11%

TLC = Thin Layer Chromatography, min. = minimal detected content, max. = maximal detected content, mid. = average detected content

### Substandard drugs in the licensed and illicit anti-malarial drug markets

Overall, 47/77 (61.0%) anti-malarial drug samples were purchased at the licensed market and 30/77 (39.0%) at the illicit market. 5/47 (10.6%) anti-malarial drug samples from licensed and 27/30 (90.0%) samples from illicit sources respectively were found to be of substandard quality. Due to signs of decline of tablets, one artesunate sample in our study was found to be substandard. The artesunate sample was labelled with the letters "not for sale" and purchased at a market place in Ougadougou.

The visual inspection was failed by 27/30 (90.0%) anti-malarial drug samples from the illicit market, while one sample purchased in the licensed market did not pass. Table 5 provides detailed information on the reasons for not passing the visual inspection differentiated by the two different market types. All products that failed the disintegration (4/77, 5.2%) or colour reaction (1/77, 1.3%) were purchased in the illicit market. Substandard concentrations of the active ingredient were detected in 5/29 (17.2%) and 4/47 (8.5%) of anti-malarial samples from the illicit and licensed markets respectively.

**Table 5:** Reasons for not passing the visual inspection separated by different kind of markets

	**Licensed market**	**Illicit market**	**Total**
**Manufacture not identified**	0	14	14
Signs of decline*	0	5	5
**Manufacture not identified + missing tablets**	0	1	1
Manufacture not identified + signs of decline*	0	5	5
missing tablets in blister + signs of decline*	1	0	1
Manufacture not identified + expired + signs of decline*	0	1	1
Manufacture not identified + package damaged + signs of decline*	0	1	1
**Total**	1	27	28

Signs of decline = sticky, broken, dirty, or high abrasion

## Discussion

In Burkina Faso, ACT is recommended for the treatment of uncomplicated malaria since 2005, but until recently, these drugs were not available through governmental health services [2]. Also private pharmacies in this country reported difficulties in the constant supply with ACT, due to shortages at the wholesalers. Increasing demand, accompanied by unavailability through legal sources, will be likely to contribute to the growing problem of fake medications [11]. Beside this, criminal greed, the lack of legislation, lack of law enforcement, corruption, and complex trade arrangements, are facilitating the influx of substandard and counterfeit products in the developing world [11].

The main result from this study is the large proportion of anti-malarial drugs found to be substandard in Burkina Faso. Nearly half of the sample showed varying degrees of impaired quality and, probably not surprising, the great majority of those were from the illicit market. However, all failures detected in nevertheless which step of the quality testing procedure were treated alike in our analysis. This is despite the fact that the different types of reduced quality may well have different impacts (e.g. a not correctly labelled and/or sticky, broken and dirty tablet might have contained the proper amount of active ingredient, and thus would not have necessarily reduced treatment success). Nevertheless, international drug quality standards should be applied worldwide.

Our results support the findings from other studies which have addressed the topic of substandard drugs in SSA [9,26-35]. In a study from Cameroon for example, 32% of chloroquine, 10% of quinine, and 13% of sulphadoxine/phyrimethamine samples were found to be substandard [9]. In a study on private pharmacies in Nigeria, 48% of anti-infective drugs, including anti-malarial drugs, were found to be of impaired quality [32].

**Figure 2:**
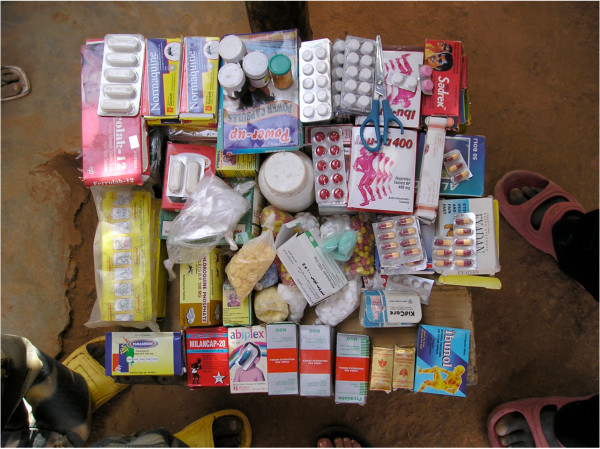
Drugs sold at an illicit market of Burkina Faso.

In the illicit market, drugs are sold as blister packs, as single tablets cut off from a blister, or just taken from big containers or plastic bags without any labelling, packaging or instruction leaflets (Figure 2). A large proportion of anti-malarial drugs obtained in the illicit market failed the visual inspection due to signs of decline and destruction. This is partly explained by tablets being carried around in huge bags from one market to the other. The substandard physical composition of products from illegal drug sources was also confirmed by the results of the disintegration test, which is most likely related to poor storage conditions. High temperatures and humidity can influence the dissolution rate, thus leading to suboptimal activity of the drug due to reduced bio-availability [30,32,36,37]. Another reason that can prevent proper disintegration is related to the formulation of the drug itself [36]. Deliberately counterfeit drugs might contain substances such as flour, baking powder or other substances. Poor disintegration is considered an important, but neglected problem in the field of drug quality [30].

**Figure 3:**
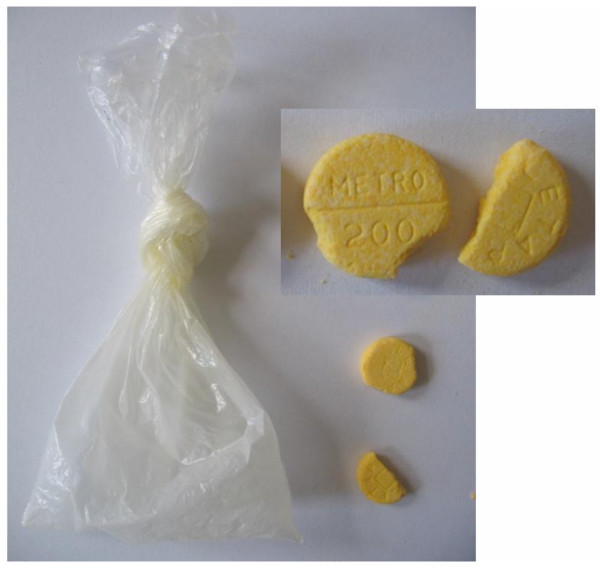
Tablets purchased as sulphadoxine/pyrimethamine in a shop.

For one product tested in this study, the presence of the stated active ingredient could not be confirmed. These tablets have been obtained in a shop in Nouna town; they came from a plastic bag without label and were sold as sulphadoxine/phyrimethamine. As the letters METRO were engraved into the tablets, these tablets were most likely metronidazol (Figure 3). This case exemplifies the problem of unqualified drug selling practices in the illicit market, as it has been reported also from neighbouring countries, too [23].

Although most substandard anti-malarials detected in this study were found in the illicit market, some were also found in the licensed market. The majority of these were quinine tablets. Currently we cannot fully exclude that these results might be linked to a methodological failure, as other quinine samples of the same brand were found to be of good quality. However, as results were confirmed in the reference laboratory, it was concluded, that some of the quinine tablets were of substandard concentrations of the active ingredient. As a consequence, a larger sample should be tested in the near future for validation. The fact that different samples of the same product passed or failed can probably be explained by the use of uncontrolled, substandard active ingredients during manufacture or poor manufacturing processes. Nevertheless, to distinguish if this was performed deliberately or unintentionally will be difficult [32]. It has also been reported that sometimes poor and good quality tablets are mixed in same batch, and even tablets in blister packs have been found to be of mixed standards [8].

The sampling of this study was based on a large and representative household survey in the frame of a malaria control intervention study which was conducted in the whole NHD in early 2006. The results can thus be considered as representative for this area of rural West Africa. All drug quality testing in the field was conducted through one experienced laboratory scientist (SD) supervised by the main investigator (MT), and the confirmation tests were done in a reference laboratory of Germany. Thus, the validity of this study can be considered as high. As artemisinin drugs and ACT were not available at the time of the study in the NHD apart from private pharmacies in Nouna town, a convenience sample of these important drugs was collected in Ouagadougou, the major town of Burkina Faso. However, as the overall number of drug samples by drug class tested was rather small, results have to be interpreted with caution.

It is reassuring, that of the artemisinin and ACT drugs collected and tested only one has been found to be substandard. This product was most likely diverted from the licensed market to the illicit market, a procedure frequently observed in malaria endemic countries [8]. However, it is likely that the increasing demand for artemisinin and ACT drugs will be accompanied by increasing production and distribution of substandard and fake products in the near future [38]. There is already evidence for such a development from a study conducted in East and Central Africa on the quality of artemisinin and ACT drugs available at private pharmacies [39]. Reports from Tanzania and Cameroon also indicated the presence of counterfeit dihydroartemisinin and counterfeit artesunate in the African market [38]. As a consequence, appropriate anti-malarial drug surveillance needs to become established in all countries of SSA. In addition, governmental drug control authorities need to be strengthened, access to affordable, quality controlled drugs needs to be improved, and the population should become better informed on the risks associated with buying drugs at the illicit market.

## Competing interests

The authors declare that they have no competing interests.

## Authors' contributions

All authors read and approved the final manuscript.

MT Conception and design of the study, sample preparation, data collection, analysis and interpretation of data, drafting of manuscript, production of final manuscript.

SD Sample preparation, data collection and interpretation of data, production of final manuscript.

DS Sample preparation and data collection, production of final manuscript

BC Conception and design of the study, supervision of laboratory work, production of final manuscript.

THT Sample preparation and data collection, production of final manuscript.

AS Conception and design of the study, supervision of the study project, production of final manuscript.

OM Conception and design of the study, supervision of the study project, analysis and interpretation of data, drafting of manuscript, production of final manuscript.
